# Low-Powered View, High-Powered Mind: A Case Report of Morpheaform Basal Cell Carcinoma Mistaken for Metastatic Breast Carcinoma

**DOI:** 10.7759/cureus.54174

**Published:** 2024-02-14

**Authors:** Riley K Imlay, Cristhian Perez Torrico, Abdullahi Sulaimann, Zachary M Grimes, Shane Monnett

**Affiliations:** 1 Surgery, West Virginia University School of Medicine, Charleston, USA; 2 Pathology, West Virginia University, Morgantown, USA; 3 Pathology, Charleston Area Medical Center, Charleston, USA; 4 General Surgery, Charleston Area Medical Center/West Virginia University, Charleston, USA

**Keywords:** skin cancers, potential pitfall for misdiagnosis, immunohistochemistry staining, metastatic breast carcinoma, morphea basal cell carcinoma

## Abstract

Basal cell carcinoma (BCC) is one of the most common skin malignancies worldwide. Morpheaform basal cell carcinoma (MBCC) is a rare aggressive subtype of BCC that presents with unique histologic features. Both are treated surgically and have an excellent survival rate. Metastatic breast carcinoma, on the other hand, has a poor survival rate along with a more burdensome therapeutic route including chemotherapy. Due to an overlap in common immunohistochemistry stains, there is a possibility of confusing the diagnosis of BCC with metastatic breast carcinoma resulting in potential patient harm. Therefore, a timely and accurate diagnosis distinguishing these malignancies is essential. We report a near-miss event in which a 77-year-old female with MBCC was mistakenly diagnosed with metastatic breast carcinoma. We discuss the details of these stains, characteristic features of MBCC, and treatment options and emphasize the importance of combining laboratory medicine with clinical expertise to improve patient outcomes.

## Introduction

Basal cell carcinoma (BCC) is a steadily increasing malignancy worldwide with approximately 4.3 million cases diagnosed annually in the United States [1]. BCC primarily affects photoexposed areas of the body with around 80% appearing on the head, half of these manifesting on the cheeks and the nose [2]. Morpheaform BCC (MBCC) is a rare aggressive subtype of BCC, accounting for only 5%-10% of all BCCs [1]. The risk of development in a Caucasian individual varies between 33% and 39% for men and 23% and 28% for women [2]. MBCC is most seen on the face and neck and is linked with high recurrence rates and more severe tissue destruction [1]. Traditional treatment methods for BCC and its subtypes include Mohs micrographic surgery, cryotherapy, electrosurgery and curettage, radiotherapy intralesional, topical chemotherapy, immunotherapy, photodynamic therapy, and surgical excision [3].

We report a 77-year-old female presenting with a back lesion initially diagnosed as metastatic carcinoma of mammary origin (stage IV breast cancer); however, upon clinical evaluation and re-testing, the lesion was identified as MBCC. Given the drastic differences in patient therapeutic options and outcomes, a timely and accurate diagnosis distinguishing these malignancies is essential. We discuss the differences and similarities between the pathology stains that led to this near-miss event and emphasize the importance of healthcare workers conducting a thorough history and physical exam in conjunction with lab and imaging results.

## Case presentation

**Figure 1:**
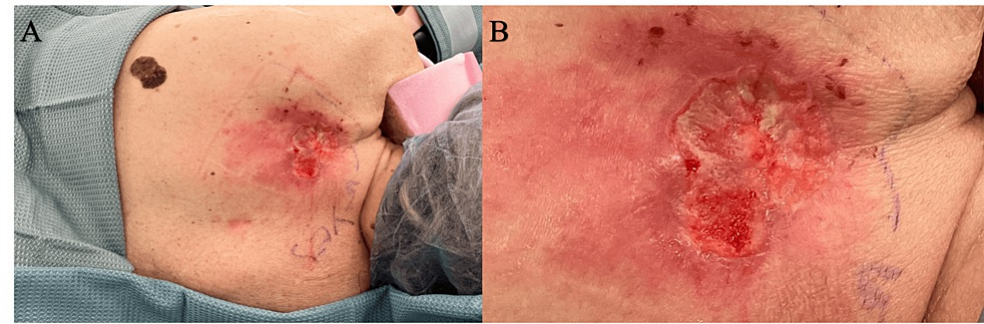
Photograph of open fungating ulcer (A) A 77-year-old female patient presents to the breast clinic with a fungating ulcer on the posterior left shoulder. (B) An enhanced image of the lesion reveals rolled edges and telangiectasias throughout, characteristics suggestive of basal cell carcinoma.

A 77-year-old Caucasian female patient presented to the breast clinic with an open fungating ulcer on her posterior left shoulder for at least two months. She reported having a "mole" in that area for years prior and that it had recently transformed into this open scabbing wound (Figure 1). On physical exam, the ulcer was noted to measure 3 cm in diameter with rolled edges and telangiectasias, characteristics suggestive of BCC. Significantly, the patient’s breast exam bilaterally revealed no dominant masses, peau d'orange, skin dimpling, or nipple retraction and discharge. There was no supraclavicular, infraclavicular, or axillary lymphadenopathy bilaterally. The patient’s breast history included menarche occurring at age 12, regular menses until menopause at age 47, first live birth age at 18, three full-term pregnancies, and birth control and hormone replacement for only a few months. The patient had no primary or secondary relatives with breast cancer; however, a screening mammogram done one month prior indicated BI-RADS (Breast Imaging Reporting and Data System) 4 of both breasts. Pertinent past medical history was significant for stage IV chronic kidney disease (CKD), coronary artery disease with left systolic heart failure, and BCC of the left nasal ala treated with Mohs surgery with negative margins followed by a bilobed flap to left nasal ala defect with placement of alar batten graft in 2021.

Several weeks before presenting to the breast clinic, the patient visited a dermatologist out of concern for the lesion. The initial pathological evaluation of the punch biopsy indicated “concern for squamous cell carcinoma.” The pathology report of the biopsy returned with the diagnosis of “cutaneous involvement by metastatic carcinoma, with mammary origin favored,” resulting in a breast clinic referral. Considering the ulcer had already been biopsied and interpreted as metastatic breast cancer, there was no need for a breast biopsy as this automatically indicated stage IV breast cancer. And so, there was a discrepancy between the clinical exam findings and laboratory diagnosis, which would direct the patient to two very different treatment paths. If this is cancer of epithelial origin, either squamous or BCC, the patient would undergo an excision with negative margins and overall have a favorable prognosis. If this is metastatic carcinoma of breast origin, this automatically establishes the diagnosis as stage IV breast cancer, which carries a much lower survival rate along with the necessity of undergoing chemotherapy; in fact, the patient had already been scheduled for an oncology appointment.

The unsettling discrepancy between the initial pathology report and our clinical exam findings along with the potential of severe patient harm encouraged the decision to obtain another punch biopsy. This time, the pathology report returned with a diagnosis of “basal cell carcinoma with 'morpheaform' morphology [with] peripheral and deep margins involved.” We further explored the pathologic stains and histologic interpretations that led to such discrepancies between the two pathology reports.

Table 1 demonstrates the different stains utilized for both pathology specimens. When comparing the two reports, it was observed that there was significant overlap between the stains used, and their interpretation could complicate an accurate diagnosis. Therefore, the means of distinguishing between the patient’s lesion being BCC or metastatic breast carcinoma relies on the combination of clinical suspicion and laboratory interpretation of the biopsy.

**Table 1:** Comparison of stains used and results between the first and second pathology reports N/P: Not performed.

Stains	First pathology report	Second pathology report
CK7	+	+
GATA 3	+	+
AE 1/3	+	N/P
E-cadherin	+	N/P
HER2	-	N/P
Progesterone receptor	-	N/P
Estrogen receptor	-	-
GCDFP15	N/P	-
SOX10	N/P	-

**Figure 2:**
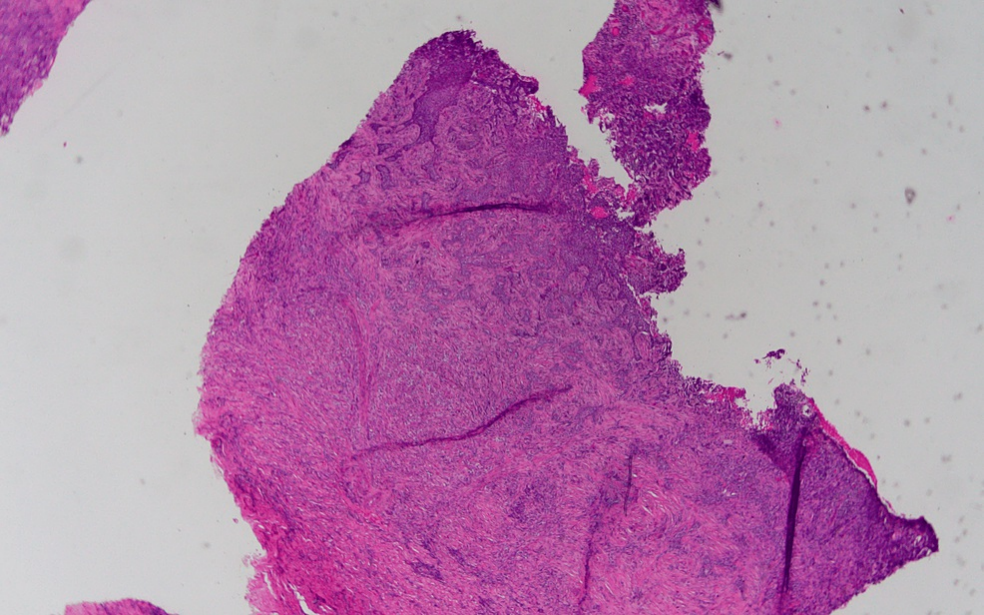
Low-power histopathological image of punch biopsy The image showing a section of the skin with surface ulceration and extension of cords of basaloid cells into the dermis.

**Figure 3:**
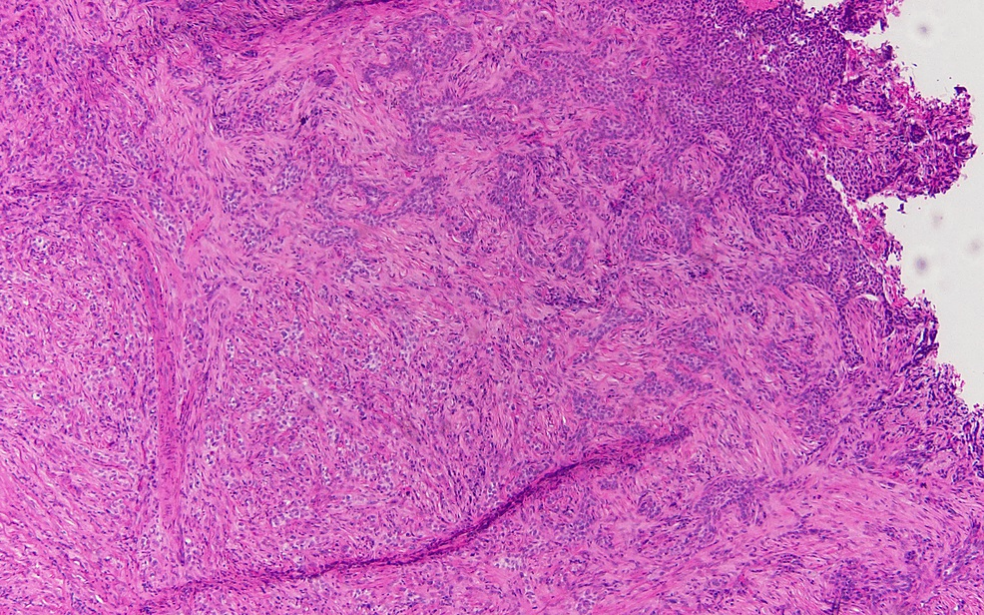
Higher magnification of histopathological imaging of punch biopsy Higher magnification shows basaloid cells with an infiltrating pattern extending into the dermis, sclerosis, and increased collagen.

**Figure 4:**
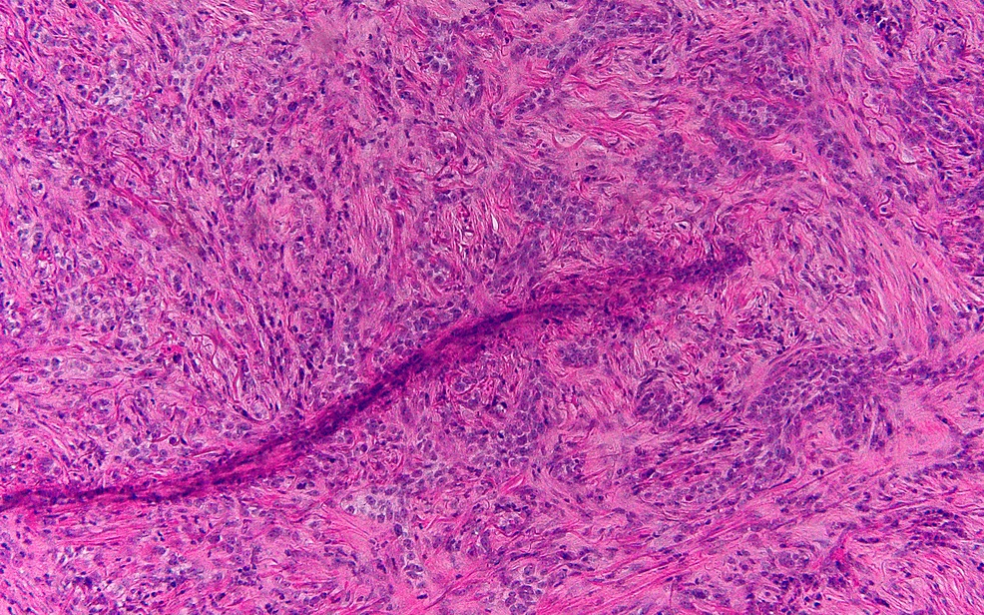
High-power histopathological imaging of punch biopsy The image shows the prominent collagen deposition and sclerotic reaction to the infiltrating lesion. The basaloid cells show some nuclear palisading and mucin production consistent with the morpheaform subtype of basal cell carcinoma.

The specimen samples analyzed during the second pathology report are illustrated in Figures 2, 3, 4. The significant detail that steers the diagnosis away from metastatic carcinoma is the fact that the basement membrane was not disrupted. If this is metastatic carcinoma, the dysplastic cells would be originating from the dermis, projecting through the basement membrane, and extending toward the epidermis. However, the dysplastic cells originate from the epithelial surface and progress downward indicating a pathology of primary skin carcinoma. As for the findings significant for MBCC, surface ulceration with an extension of cords of basaloid cells into the dermis is observed. Higher magnification reveals basaloid cells with an infiltrating pattern extending into the dermis, sclerosis, and increased collagen deposition. Importantly, the basaloid cells show some nuclear palisading and mucin production consistent with MBCC.

**Figure 5:**
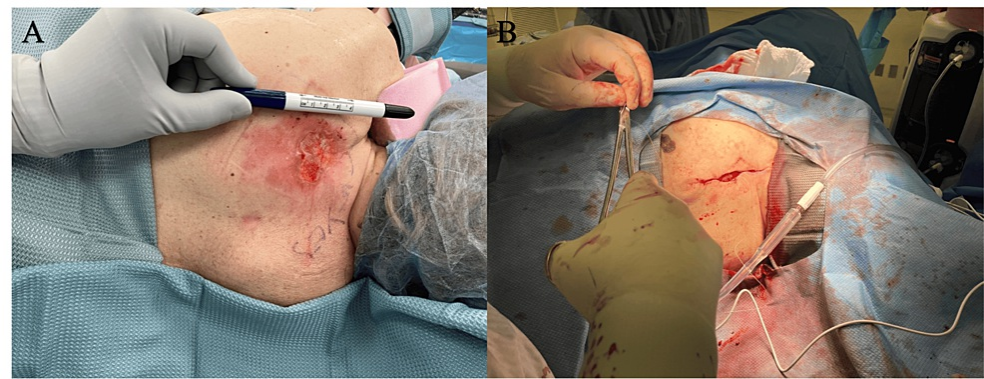
Photograph of pre- and post-excision of BCC (A) Open fungating sore measuring 3 cm in width on her posterior left shoulder present for at least two months. (B) Post-excision of the BCC, the skin was closed in a horizontal mattress fashion. BCC: Basal cell carcinoma.

Multiple factors were considered such as location, size, histological features, and the patient’s medical history to establish a treatment plan. A wide local excision of the left posterior shoulder BCC was performed with appropriate margins measuring a total of 12 x 6 cm, and fasciocutaneous flaps were raised medially and laterally to mobilize the skin edges for primary closure. The patient was scheduled for a two-week follow-up during which the pathology report returned confirming negative margins. Figure 5 illustrates the surgical process.

Given her prior history of BCC, the aggressive nature of MBCC, the 3 cm diameter of her lesion, and the treatment decision of surgical excision, this patient should be closely monitored for recurrence or metastasis of her cancer. However, this is still a much more favorable and benign treatment course compared to her original diagnosis of metastatic breast carcinoma.

## Discussion

There have been at least 26 subtypes of BCC described in the literature [3]; therefore, an accurate diagnosis is essential to direct treatment. As described by Dr. Henry Randle, “MBCC is a subtype of BCC comprised of thin strands of basaloid cells that tend to grow between the collagen and hair follicles, sweat glands, cartilage, bone, nerves, and vessels, usually invading the dermis or subcutaneous tissue deeply. MBCCs contain a high number of type IV collagenase which gives the malignancy the capacity to degrade basement membranes, therefore allowing the tumor to invade host tissue, form irregular borders, and metastasize” [3].

In the present case, two different punch biopsies of the patient’s shoulder lesion resulted in conflicting pathology reports, which would lead to very different prognoses and patient care decisions. According to data, the five-year relative survival rates after BCC diagnosis and squamous cell carcinoma (SCC) are close to 100% and 90%, respectively [4]. The 10-year actuarial breast cancer-specific survival rate is 15.7% for women aged 40 and below, 14.9% for women aged 41-50, and 11.7% for women aged 51-70 [5]. The diagnosis from the original pathology report read “cutaneous involvement by metastatic carcinoma with mammary origin favored,” while the second pathology report read “basal cell carcinoma with ‘morpheaform’ morphology [with] peripheral and deep margins involved.” So, we now explored the stains and potential reasons for the different interpretations of the biopsied specimen. While discussing the differences in immunohistochemical markers used, the vital question to keep in mind is whether the tumor is of skin (primary) or mammary (metastatic) origin.

CK7 is generally expressed (with some variation) in adenocarcinoma of the lung, breast, thyroid, endometrium, cervix, ovary, salivary gland, upper GI tract, urothelial carcinoma, papillary renal cell carcinoma, and Paget disease; however, it is also a membranous and cytoplasmic marker with expression in many normal epithelia and epithelial tumors [6]. Of note, CK7 is generally negative in squamous cell carcinoma [6]. GATA3 is commonly used to identify the luminal differentiation of breast epithelium, development of the urothelium, and trophoblastic differentiation but can also be positive in skin and breast pathologies [7]. AE 1/3 is a mixture of two different clones of anticytokeratin monoclonal antibodies (AE1 and AE3), which functions as a marker for cytokeratins 1-8, 10, 14-16, and 19; this is another non-specific marker where immunoreactivity is observed in epithelia and most carcinomas (tumors of epithelial origin) [8]. E-cadherin is a transmembrane protein that functions in cellular adhesion which, in the context of breast pathology, is commonly used to differentiate lobular carcinoma in situ (stains negative) from ductal carcinoma in situ (stains positive); however, E-cadherin can be positive in any carcinoma of epithelial origin, including BCC [9].

The second report shares three of the same stains but differentiates in the use of GCDFP15 (negative) and SOX10 (negative). GCDFP15 is commonly used to differentiate breast carcinoma metastatic to ovary (positive) from primary ovarian carcinoma (negative) [10]. In the context of breast pathology, a positive stain of GCDFP15 indicates a potential for lobular breast carcinoma (90%), primary breast carcinoma (72%), and metastatic breast carcinoma (80%) [10]. As the specimen stained negative, both metastatic and primary breast carcinoma would seem unlikely. SOX10 is a transcription factor known to be crucial in the specification of the neural crest as well as the maintenance of Schwann cells and melanocytes. It is expressed in the nuclei of melanocytes and breast myoepithelial cells; thus, it is commonly positive in breast basal-like, triple-negative, and metaplastic carcinoma [11].

Therefore, by reviewing the stains used to determine the most likely diagnosis in this patient, one can appreciate that many of the stains are sensitive but extremely non-specific. Therefore, the combination of the patient’s clinical examination, interpretation of stains used, and histopathological findings supports the diagnosis of BCC over metastatic breast carcinoma.

Treatment

The primary factors determining whether a patient with BCC is at high risk for recurrence or metastasis are explained in the following [3].

Location

Ear and periorbital lesions have the highest recurrence rates.

Size

As the diameter of the lesion increases, the rate of recurrence also increases. The five-year recurrence rate of a primary BCC on the head was 3.2% for lesions less than 0.5 cm, 8% for lesions 0.6-1 cm, and 9% for lesions larger than 1 cm.

Histologic Features

There are aggressive (morpheaform, micronodular, metatypical) and nonaggressive (nodular, superficial) subtypes of BCC. The nonaggressive subtypes tend to have fewer positive margins and recurrence rates of 1%-6%, while the aggressive subtypes have increased rates of positive margins and recurrence rates of 30%. In particular, the morpheaform subtype has been reported to extend 7.2 mm beyond the estimated borders compared to 2.1 mm for the nodular subtype; therefore, the traditional recommendation to remove 4-5 mm of normal-appearing skin surrounding the BCC should be increased in the morpheaform subtype.

Type of Treatment

Traditional treatment methods for BCC include surgical excision, radiotherapy, electrosurgery, curettage, intralesional and topical chemotherapy, immunotherapy, and photodynamic therapy. Mohs microsurgery is commonly accepted as the most effective means of removing BCC with cure rates around 97%.

Previous Treatment

Primary BCCs have a five-year recurrence rate of 10.6%, whereas previously treated BCCs have a rate of 15.4%. All these factors should be considered when deciding the treatment options. The current patient is a 77-year-old Caucasian female with a left posterior shoulder lesion that has been growing for the last two months. She had a biopsy performed on the 3 cm lesion that was consistent with MBCC. The patient’s past medical history was significant for BCC of the left nasal ala, which was treated with Mohs surgery with negative margins in 2021. As established, Mohs surgery is considered the gold standard for removing BCC; however, the process is very time intensive requiring hours of anesthesia, which would be taxing on a patient with stage IV CKD and left systolic heart failure. For these reasons, the patient underwent a wide local excision with appropriate margins.

## Conclusions

BCC is an ever-increasing malignancy worldwide encompassing numerous subtypes, including the rare and aggressive MBCC. These malignancies are treated surgically with high rates of survival. Conversely, metastatic breast carcinoma has a much lower survival rate along with more intensive treatment including chemotherapy. Even with the innumerable benefits of modern laboratory medicine, clinicians must utilize their expertise to assist in a diagnosis based on the features of the pathology encountered. With malignancies as common as BCC, practicing physicians must be able to recognize the common characteristics and guide treatment accordingly. Staining methods for BCC and metastatic breast carcinoma have the potential to overlap and therefore may lead to misdiagnoses and adverse patient outcomes. Clinicians and pathologists should be made aware of this reality to properly guide treatment and improve patient care.
